# Editorial: Lifestyle interventions for childhood obesity: Broadening the reach and scope of impact

**DOI:** 10.3389/fendo.2022.1099534

**Published:** 2022-12-01

**Authors:** Jared M. Tucker, Domenico Corica, Seema Kumar

**Affiliations:** ^1^ Health Optimization Services, Helen DeVos Children’s Hospital, Grand Rapids, MI, United States; ^2^ Department of Pediatrics and Human Development, College of Human Medicine, Michigan State University, Grand Rapids, MI, United States; ^3^ Department of Human Pathology of Adulthood and Childhood, University of Messina, Messina, Italy; ^4^ Division of Pediatric Endocrinology, Department of Pediatric and Adolescent Medicine, Mayo Clinic, Rochester, MN, United States

**Keywords:** pediatric weight management, health behavior, nutrition, culinary education, physical activity, cardiometabolic health, parent education, prevention

Childhood obesity is a growing epidemic and is associated with several physiological and psychological detrimental effects (1, 2). Improving lifestyle behaviors, including dietary habits, physical activity, sedentary behavior, and sleep remain the cornerstone of childhood obesity treatment (3). However, pediatric weight management (PWM) and prevention practices vary widely in treatment focus and delivery methods as programs seek to elucidate best practices for children’s weight and health outcomes (4).

This Research Topic includes six studies that explore innovative strategies for preventing and treating pediatric obesity and novel markers for assessing improvements in obesity-related health risks. Specifically, one study seeks to better understand the interactions between social determinants of health, obesity, and related health behaviors (Seum et al.). Three studies evaluate the feasibility and impact of novel interventions for obesity prevention and treatment (Schmit et al., Tucker et al., Cao et al.), and two studies aim to provide a better understanding of obesity-associated health outcomes (Bhangoo et al., Jørgensen, et al.).

The development of childhood obesity is complex and multifactorial, and include genetic, epigenetic behavioral and psychological influences, which are further shaped by social and environmental factors. Thus, a better understanding of how these facets interact is key to effective obesity prevention and treatment strategies. Seum et al.explored associations between parental education, an indicator of socio-economic status, and body mass index (BMI) changes over time in a nationwide sample of German youth. As hypothesized, lower parental education was associated with higher BMI over a 5-year period. Furthermore, several health behaviors were identified as partial mediators, including breakfast consumption and total screen time, suggesting less frequent breakfast consumption and greater screen time use among youth in households with lower parental education. These results indicate a need for targeted behavioral strategies among households with less education, a key finding for many PWM programs who primarily serve such families.

Promotion of healthy nutrition behaviors often focuses on what to eat, while building the knowledge and skills for how to change eating habits often receives insufficient attention. Culinary education seeks to address these potential deficiencies by providing hands-on learning and skill building through healthy meal preparation. Schmit et al. evaluated nutrition and culinary changes in 180 predominantly Hispanic/Latino youth who participated in a weekly, after-school culinary education program known as CHEF Bites. Program participation and satisfaction was high, and participants demonstrated meaningful increases in nutrition knowledge and culinary skills as well as improvements in some eating behaviors. These results are promising and are a valuable component in building the knowledge and skills youth need to make healthier nutritional choices.

Nutritional interventions are critical to PWM, but adoption and long-term adherence is often difficult. Time-limited eating, where calorie consumption is restricted to a particular number of consecutive hours during the day, has shown promise in adults but remains largely untested in youth. Tucker et al. evaluated the acceptability of time-limited eating among over 200 youth enrolled across five PWM programs and found that up to two-thirds of parents and more than half of youth surveyed were interested in time-limited eating in some form. Most barriers identified by families involved work and school schedules, as well as snacking and hunger; however, if families are able to overcome these challenges, time-limited eating appears to be a feasible option for many youths in PWM treatment.

Physical activity promotion is a crucial treatment component of PWM as it not only impacts obesity but independently reduces cardiometabolic health risks. High-intensity interval training (HIIT) mirrors children’s tendency to actively play in intermittent bursts and studies have demonstrated high adherence, yet its effects on glycolipid metabolism remains mixed. Cao et al. conducted a meta-analyses evaluating the impact of HIIT on glycolipid metabolism compared to non-training controls in youth with metabolic disorders, and found beneficial effects on triglycerides (TG), total cholesterol, low-density lipoprotein (LDL), high-density lipoprotein (HDL), blood glucose, blood insulin, and homeostasis model assessment (HOMA)-IR. Health benefits were similar to moderate-intensity training, though HIIT was more time-efficient. Based on these positive findings, future research is warranted to evaluate of the acceptability and effectiveness of HIIT in PWM.

Insulin resistance in children with obesity precedes development of type 2 diabetes (T2DM) and metabolic syndrome. Timely diagnosis and treatment of insulin resistance (IR) is essential as treatment at an early stage may prevent T2DM and dyslipidemia. Bhangoo et al., in a cross-sectional pilot study of multiethnic youth, demonstrated an inverse correlation between IGFBP-1 levels and indices of adiposity and traditional markers of IR such as HOMA, TG/HDL and TG. More importantly, fasting IGFBP-1 levels were lower in adolescents with overweight in comparison to children with obesity despite no differences in the traditional markers of IR. Larger, longitudinal studies in children would shed light on the utility of IGFBP-1 as an independent marker of IR that might be helpful in its early detection.

An association between serum uric acid (SUA) and degree of adiposity has been demonstrated in adults and SUA has been shown to be an independent predictor of T2DM and metabolic syndrome. This relationship has not been studied systematically in children. Jørgensen, et al. demonstrated a positive association between SUA and degree of adiposity in children. Furthermore, SUA decreased in children with weight loss while participating in a multifactorial lifestyle intervention and, in contrast, increased in those with weight gain after an average follow-up of almost 2 years. Additional studies are warranted to examine if hyperuricemia contributes to metabolic syndrome or if hyperuricemia is a consequence of IR. These data would be potentially helpful in early dentification of children at increased risk for cardiometabolic disease.

The studies in this Research Topic provide valuable and practical information for clinicians working to improve lifestyle interventions for the prevention and treatment of obesity and for health professionals seeking to improve evaluations of obesity-related outcomes. In addition, this novel work illuminates future research opportunities that will continue to expand our understanding of best practices for combating pediatric obesity.

## Author contributions

All authors planned the outline of the editorial. JT and SK drafted the manuscript. All authors contributed to manuscript revisions and approved the final version.

## Conflict of interest

The authors declare that the research was conducted in the absence of any commercial or financial relationships that could be construed as a potential conflict of interest.

## Publisher’s note

All claims expressed in this article are solely those of the authors and do not necessarily represent those of their affiliated organizations, or those of the publisher, the editors and the reviewers. Any product that may be evaluated in this article, or claim that may be made by its manufacturer, is not guaranteed or endorsed by the publisher.
